# F166. LOW-GRADE INFLAMMATION IN FIRST-EPISODE PSYCHOSIS IS DETERMINED BY WAIST CIRCUMFERENCE INCREASE

**DOI:** 10.1093/schbul/sby017.697

**Published:** 2018-04-01

**Authors:** Jaakko Keinänen, Outi Mantere, Tuula Kieseppä, Maija Lindgren, Teemu Mäntylä, Eva Rikandi, Jouko Sundvall, Minna Torniainen-Holm, Jaana Suvisaari

**Affiliations:** 1 University of Helsinki; 2 McGill University; 3 University of Helsinki, Helsinki University Hospital; 4 National Institute for Health and Welfare

## Abstract

**Background:**

There is evidence of low-grade inflammation in psychosis, as measured by the high-sensitivity C-reactive protein (hs-CRP). Significant weight gain is common during the first months of antipsychotic treatment. In the general population, overweight and obesity often lead to systemic low-grade inflammation. Lifestyle factors, such as smoking, can contribute to the pro-inflammatory changes. The metabolic changes in people with first-episode psychosis (FEP) taking place after the onset of psychosis can be especially harmful as these individuals are typically young and without major somatic illnesses. We aimed to study how the low-grade inflammation, measured by hs-CRP, develops in FEP and to clarify the effect of waist circumference increase in the inflammation.

**Methods:**

The Helsinki Early Psychosis Study recruited FEP patients (age 18 to 40 years) attaining their first treatment for psychosis from the catchment area of the Helsinki University Hospital. We recruited 95 FEP patients and 62 controls. The inclusion criterion for the study was receiving a score of at least 4 in Unusual thought content or Hallucinations in the Brief Psychiatric Rating Scale - Extended (BPRS-E). Diagnoses of psychotic disorders according to the DSM-IV criteria were later verified using the Structured Diagnostic Interview for DSM-IV and reviewing all medical records. Substance-induced psychotic disorders and psychotic disorders due to a general medical condition were excluded. We measured the changes in hs-CRP, weight, waist circumference, glucose metabolism and lipids at baseline and at follow-ups of 2 and 12 months. We used linear mixed effects models to analyze the relationship between hs-CRP and waist circumference. In the model, we included a random intercept for each patient and, as fixed effects, we entered sex, time (days from baseline measurement), waist circumference and antipsychotic use at each assessment point, and baseline cigarette smoking.

**Results:**

At baseline, FEP patients (mean age 26.1 years) had higher insulin resistance, total and LDL cholesterol, apolipoprotein B and triglyceride levels than controls. However, baseline weight and waist circumference, hs-CRP, fasting glucose and HDL cholesterol were similar between patients and controls. A robust change in anthropometric measures and inflammation was evident among patients by 12 months. Hs-CRP was significantly higher in patients at 12-month follow up than at baseline (baseline hs-CRP 0.67 mg/l, IQR 0.33–2.54; 12-month 1.73 mg/l, IQR 0.49–4.21; Wilcoxon signed-rank p = 0.007). When at the baseline the prevalence of overweight or obesity was 30% (28/94) in patients with FEP, by 12 months the prevalence was 59% (35/59) (McNemar′s test p < 0.001). The proportion of patients gaining ≥ 7 % of baseline weight was 68 % (40/59). The median weight gain among patients was 9.6 kg (IQR 1.5–13.6 kg), and the waist circumference increase 6.0 cm (IQR 2.0–13.0 cm). In the mixed effects model waist circumference (p < 0.0001) and sex (p = 0.014) were significantly associated with hs-CRP level.

**Discussion:**

We detected a significant elevation in hs-CRP in people with FEP during the first treatment year. The rise in hs-CRP was determined by waist circumference increase. Patients with FEP are in a marked risk of developing abdominal obesity and subsequent low-grade inflammation during the first year of treatment. Prevention of the early metabolic changes in first-episode psychosis is important, as abdominal obesity and inflammation are associated with increased risk of cardiovascular events and mortality.

